# Multitasking NPOs: An Analysis of the Relationship Between Funding Intentions and Nonprofit Capacities

**DOI:** 10.1007/s11266-021-00364-4

**Published:** 2021-06-18

**Authors:** Sara Stühlinger, Sophie E. Hersberger-Langloh

**Affiliations:** Center for Philanthropy Studies (CEPS), University of Basel, Steinengraben 22, 4051 Basel, Switzerland

**Keywords:** Benefits theory, Multitasking theory, Capacity building, Nonprofit organizations

## Abstract

Nonprofit organizations (NPOs) often find themselves under pressure to invest all of their available income in mission-related activities rather than in capacity building. We investigate one factor that can influence the decision to invest in such capacity-building tasks: funding sources pursued by an organization. Drawing on the benefits theory of nonprofit finance, we take these funding sources as predetermined by an organization’s mission and propose an extension of the theory by linking it to economic multitasking theory, which states that organizations prioritize tasks that offer greater and more measurable rewards. Through regression analyses of survey data from Swiss nonprofits, we analyze the extent to which funding sources sought affect the amount of effort invested in three areas of capacity building: public relations, impact focus, and resource attraction parameters. The results support the predictions of multitasking theory by showing that the effort invested in certain capacity-building tasks is affected considerably by seeking a specific funding source. The effects are stronger for resource attraction-related tasks than for tasks closer to the service delivery of NPOs. The results indicate that an organization’s mission affects not only the available funding sources but also the extent to which an organization invests in its capacities, which can lead to a ‘lock-in’ status for organizations.

## Introduction

Nonprofit organizations (NPOs) face challenges that are inherent to the idiosyncrasies of this type of institution. One vital challenge for NPOs is capacity building. Although funding has decreased in recent decades, demand for NPO services has increased (Kahnweiler, 2011). As a result, such organizations have to operate on limited resources. Furthermore, when investing in capacities, NPOs must consider and manage their complex stakeholder structures based on stakeholders’ different expectations of them (Balser & McClusky, 2005). For example, nonprofits are under pressure to invest a high share of the funds they receive directly into their mission to keep overhead costs low (Gregory & Howard, 2009; Lecy & Searing, 2015), which especially puts administrative and fundraising capacities under additional scrutiny. However, many nonprofit capacities are positively correlated with nonprofit effectiveness (Shumate et al., 2017), i.e., how well an organization is able to achieve its mission. For NPOs, mission fulfillment is not just the ultimate goal but also serves as a long-term strategy, setting the rules for an organization’s development (McDonald, 2007). The importance of the mission and its influence on an organization’s behavior is also discussed by the benefits theory of nonprofit finance (subsequently shortened to benefits theory), which states that the source of an organization’s financing depends on its purpose (Wilsker & Young, 2010). According to this theory, the kinds of goods an NPO produces—which are inherent to its mission—influence the funding sources available to it. Consequently, two factors are determined from the outset: the organization’s purpose and its funding sources, thereby creating, to some extent, a barrier to organizational growth with NPOs being “locked in” by environmental or organizational constraints that dictate their development (von Schnurbein, 2017).

In this study, we propose a theoretical framework that extends the influence of the mission beyond the funding sources sought to nonprofit capacities. We test the effect of funding sources sought on capacity building, as nonprofit capacities are important for the development of an organization and the intention to seek funding from a specific source is a strategic decision made in an organization. It is therefore important to better understand the drivers of capacity building. To address this issue, we raise the following research question: does the type of funding an organization seeks affect its efforts invested in capacity building?

In this exploratory study, we draw on the benefits theory of nonprofit finance and multitasking agency theory to explore the funding intentions of organizations and how these intentions affect the extent to which organizations devote themselves to different types of capacities. We do this by recourse to ordinary least square (OLS) regressions with survey data collected from Swiss NPOs.

The article begins with a review of relevant theories and the introduction of our research framework, in which we explain the relationship between the mission and funding sources and how they might affect capacity building. We then provide an overview of how our data were collected and analyzed using OLS regressions. The paper concludes with a discussion of the findings and of implications for future research and practice.

## Theoretical Framework

Researchers and practitioners alike emphasize the importance of investments in organizational capacities (capacity building) (Faulk & Stewart, 2017), thereby enhancing an organization's ability to fulfill its mission by means of targeted investments in operational areas. In light of challenges such as increased public scrutiny (Ostrander, 2007), heightened competition for scarce resources (Kerlin & Pollak, 2011), and limited room for programmatic change, it is essential to understand which factors contribute to investments in these capacities by NPOs. In the present work, we propose a theoretical framework to better understand the drivers of investment in nonprofit capacities. The framework we propose is based on the benefits theory of nonprofit finance (Young, 2017) and multitasking theory (Holmstrom & Milgrom, 1991). According to the benefits theory, an NPO’s mission determines the funding sources available to it, according to who benefits from the goods and services it offers. In line with multitasking theory, organizations invest their efforts in capacities associated with greater rewards.

**Fig. 1:**
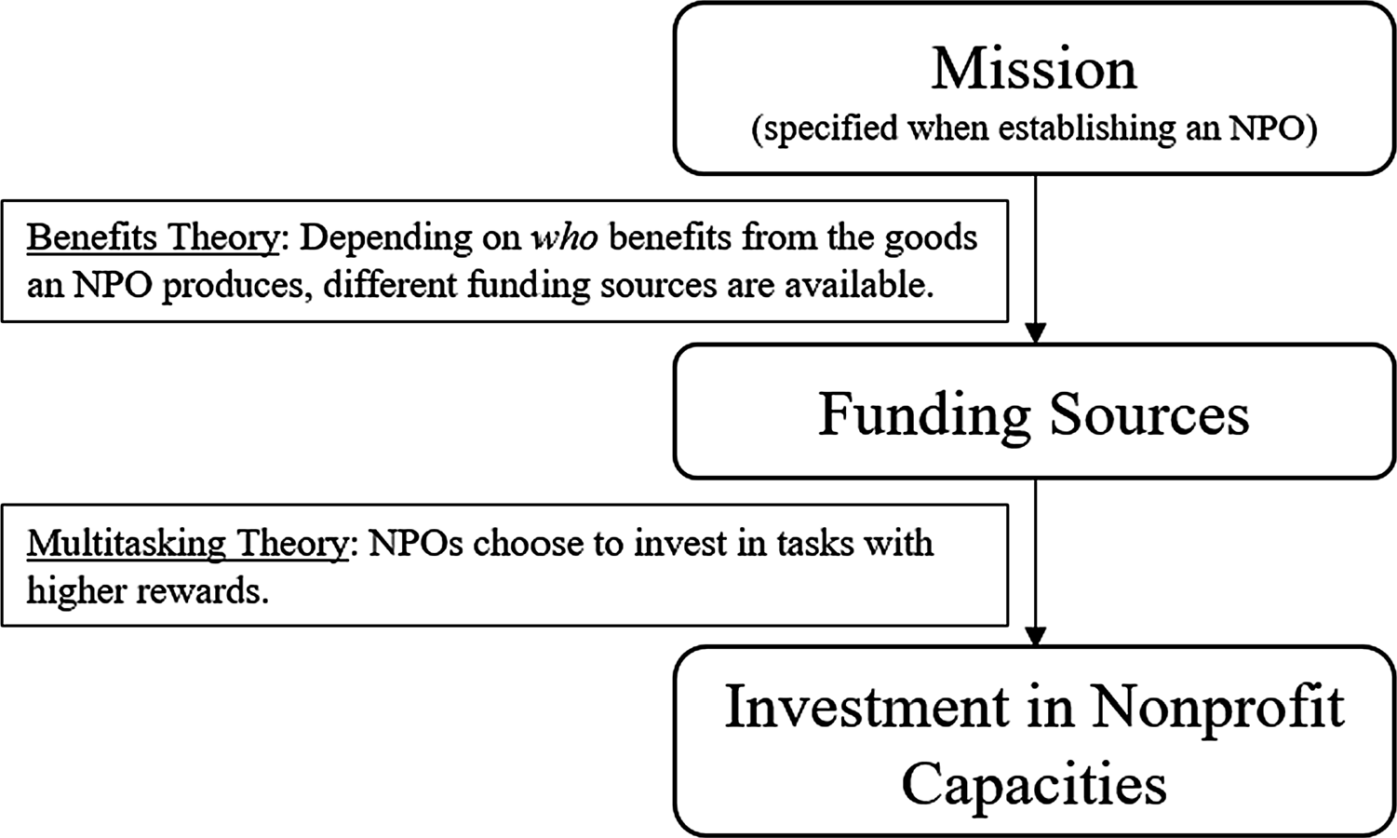
The research framework

Figure 1 depicts our research framework, which we explain in more detail in the following paragraphs:

In the following section, we first introduce the benefits theory of nonprofit finance and multitasking theory and then define nonprofit capacities to derive a research hypothesis, which we then test empirically.

### Relationship Between Mission and Funding Sources: The Benefits Theory of Nonprofit Finance

Based on the idea that incentives play a fundamental role in the financing of NPOs, Young (2007) developed a normative conceptual framework that can be regarded as the foundation of the benefits theory of nonprofit finance (first mentioned and empirically tested by Wilsker and Young (2010)). The theory states that an NPO’s funding structure is determined by the kinds of goods it provides. The authors argue that depending on their particular missions, NPOs produce various public and private goods that benefit their stakeholder groups. In return, these stakeholders support NPOs through diverse funding and financing mechanisms (Young, 2017). The sources of income should therefore be consistent with the nature of services and goods offered to the providers of these resources. According to the theory, NPOs providing public goods (such as disease prevention) are more likely to receive public funding or donations; those that provide private goods (such as nursing homes) are more likely to finance themselves through earned income. In previous research, the benefits theory has been studied empirically by exploring the funding structures of NPOs and categorizing them according to whether their services are private, public or mixed goods (see Young, 2017, pp. 46–47 for an overview).

Linking the financing of NPOs to the kind of goods or services NPOs offer emphasizes an underlying assumption: that the services an organization offers are transactional in nature. Someone (for example donors, beneficiaries, or the public sector) benefits from a product or service provided by an NPO, and depending on who benefits, different (appropriate) income sources are available to the NPO (von Schnurbein, 2017; Young, 2017). For certain types of funding and goods, this transactional nature is evident. Earned income, for example, is clearly transactional in nature: Customers are rewarded for their payment with goods or services from an NPO (Daniel & Eckerd, 2019). For other links between funding and goods, the rewards are not as obvious, but research has shown that even altruistic acts such as donating money have a reward (Andreoni, 1990; Roberts & Roberts, 2012), albeit an intangible one. Valentinov (2008), in his theory of positive transaction cost, argues that compared to traditional firms, NPOs are utility driven rather than profit driven. All transaction partners—i.e., stakeholders—must either derive utility or receive financial compensation from an exchange with an NPO.

### The Choice of Investment in Capacities: Utilizing Multitasking Theory

Based on the benefits theory of nonprofit finance described above, NPOs are limited in their choice of (available) funding sources by their defined missions. Finding funding is essential for NPOs, as many NPOs are notoriously short on resources (Drucker, 1989). Faced with the challenge of acquiring financial resources, nonprofit managers are also under pressure to direct as much effort and expenditure as possible toward the organization’s defined mission (Gregory & Howard, 2009; Lecy & Searing, 2015). NPOs operate within complex stakeholder networks (Padanyi & Gainer, 2004; Wellens & Jegers, 2014) with each stakeholder having a different perception of an NPO’s identity and a different interpretation of its organizational activities (Balser & McClusky, 2005). It is a challenge for NPOs to spend their funds and divert their efforts in such a way where all of their stakeholders are satisfied. NPOs therefore act as agents with multiple principals that place different or even conflicting demands on them (van Puyvelde et al., 2012).

According to the multitasking theory developed by economists Holmstrom and Milgrom (1991), agents focus their efforts on tasks that are measurable and rewarded, potentially at the expense of other tasks, due to time or monetary constraints (crowding out). The allocation of effort to tasks is determined by the relative benefit derived from each task. Although this theory was developed and tested on an individual level (workers as agents), it can also serve as a framework with which to predict the behavior of an organization as an agent (Dewatripont et al., 2000).

One example of this phenomenon concerns regulatory measures imposed on organizations aimed at cutting costs; such measures will lead to a reduced focus on tasks that improve quality due to the increased production cost that these tasks involve (Laffont & Tirole, 1991). Similarly, when faced with the demands of multiple stakeholders (principals) and multiple tasks to be performed, NPOs may choose to prioritize tasks that offer the greatest reward or utility.

The extent to which NPOs can fulfill tasks depends on their organizational capacities, which are therefore means to an end (Stevens, 2001). Although researchers and practitioners alike agree that such capacities play a crucial role in mission fulfillment, they remain difficult to define or operationalize (Andersson et al., 2016). Christensen and Gazley (2008) use for their analyses of organizational capacities cited in the business, public and nonprofit literature a framework encompassing infrastructure, human resources, financial resources and management systems, and the external environment. The authors base these dimensions on previous studies and understand them “as a synthesis rather than an alternative” to the concepts they use for their framework (Christensen & Gazley, 2008, p. 268). The authors find that organizational capacities are multidimensional and present a comprehensive list of how capacities are operationalized. Shumate et al. (2017) operationalize nonprofit capacities based on eight concepts: financial management, adaptive capacity, strategic planning, external communication, board leadership, operational capacity, mission orientation, and staff management. Despard (2017) finds empirical support for four dimensions of capacity building: resources, programs, board development and management capacity measured by 19 items for nonprofit human service organizations. In their study of the Canadian nonprofit sector, Hall et al. (2003) identify investments in financial and human resources and structural capacities as crucial to an organization’s ability to fulfill its mission.

Following the definition provided by Shumate et al. (2017, p. 156), we define nonprofit capacity “as the processes, practices, and people that the organization has at its disposal that enable it to produce, perform, or deploy resources to achieve its mission.” Capacity building is therefore indeed a means to an end. Some of these tasks are closely related to ‘the means’ or the resources needed to maintain operations, while others are closely related to ‘the ends,’ which in this case stand for the services and programs an organization provides for its beneficiaries to fulfill its mission. For example, among the capacities identified by Shumate et al. (2017), capacity *financial management* is closely related to attracting resources, whereas capacity *mission orientation* is directly linked to the ultimate goal of an NPO.

**Fig. 2:**
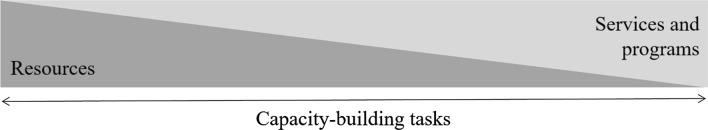
Spectrum of capacity building

This spectrum of capacity building is depicted in Fig. 2.

Capacity-building tasks along the spectrum of organizational activities (from attracting resources to serving beneficiaries) are to a certain extent mutually dependent insofar as they affect and facilitate each other and are all aimed at ensuring an organization’s survival and development. However, such tasks can be clearly distinguished in terms of the associated operations and underlying competencies needed to put them into practice. As research has shown, there is not only *one* nonprofit capacity but rather several distinct nonprofit capacities (Andersson et al., 2016; Despard, 2017; Shumate et al., 2017, among others).

### Hypothesis

In Fig. 1, we summarized the relationships between the aforementioned theories and presented our research framework. The benefits theory states that an NPO’s mission affects its composition of funding sources, since the availability of such sources depends on who benefits from the type of services offered. The first part of the framework focused on the relationship between mission and funding sources has already been empirically tested (Young, 2017) and is generally accepted. However, the second part of the proposed framework has, to our knowledge, not yet been explored in nonprofit research, which renders this research an exploratory study. Multitasking theory predicts that organizations invest their efforts in tasks associated with greater rewards. Accordingly, we expect NPOs to focus their efforts on capacity-building tasks related to their most important funding source(s).

Therefore, to investigate the extension of this theory and the relationship to investment in capacities, we formulate our research hypothesis as follows:

The funding intentions of an NPO influence the effort put into capacity-building tasks, as some tasks are more highly rewarded than others.

## Methodology

### Data

Switzerland constitutes an interesting case because it has a relatively large NPO sector in terms of its work force (Helmig et al., 2017) and its nonconclusive classification as a type of civil sector. From a social origins theory perspective, Switzerland can be assigned to both the social-democratic (Einolf, 2015) and liberal categories (Helmig et al., 2017). Although the nonprofit sector as a whole can be considered a special case within continental Europe (Helmig et al., 2017), the organizations and processes found within the sector are comparable to those of other Western countries (Helmig et al., 2011). The sector comprises approximately 100,000 organizations, including over 13,000 charitable foundations (von Schnurbein & Perez, 2018) and approximately 80,000 associations, most of which are of a charitable nature (Helmig et al., 2010). 58% of their financing comes from fees and sales, 35% comes from government funding, and 8% comes from philanthropy; however, financing structures vary widely depending on the field of activity (Helmig et al., 2017).

Accordingly, for our empirical analysis, we used survey data from Swiss NPOs from various smaller indices. Since there is no central register of NPOs in Switzerland, the exact population of NPOs and charities and their distributions across areas of activity are unknown. We therefore applied a nonprobability sample of operational organizations or NPOs that not only fund but also manage and implement their own projects. We applied typical case sampling and included cases from charities bearing a quality seal, NPOs with a focus on environmental issues, and a sample of organizations from the trade register. Based on a keyword search, we randomly selected organizations from the health and housing sectors. These subsamples allowed us to include typical cases from vital nonprofit sectors with large organizations in the overall sample. The survey, sent to 3,053 Swiss NPOs in 2018, was administered as part of a larger research project on management challenges faced in NPOs. The data were collected in Switzerland by means of a postal survey in German and French, after the questionnaire had been pretested in both languages in line with the recommendations of Hak et al. (2008). Organizations were sent the questionnaire, a cover letter explaining the overall goal of the research project, and a return envelope. Of the 622 questionnaires received (response rate of 20.4%), 544 questionnaires contained complete answers to all relevant questions.

Of these 544 organizations, 41.9% were foundations (228), and 56.9% were associations (310). On average, the organizations were founded 50 years ago, had 62 employees and had operating expenses of 12 million CHF in 2017. The responding organizations were asked to select their areas of activity (ICNPO categories). A total of 238 of the organizations selected health (44%), 211 selected social services (39%), 125 selected education and research (23%), and 125 selected culture and recreation (23%), and all other categories were selected less often (< 15%). These four categories are the most prominent in terms of employees and budget in the Swiss nonprofit sector (Helmig et al., 2010) and were thus focused on in the sampling process.

### Variables

As described above, the survey was designed with a broader scope and included questions on research projects relating to management challenges faced in NPOs, such as financial competencies or market orientation. The variables used were selected from this pool of survey items. All dependent and independent variables used concerned questions answered by the respondents on a five-point Likert scale ranging from 1 = [I agree] to no extent, to 5 = [I agree] to a large extent. We treated the data as continuous, as we assumed equal distances between the five possible response options (Hair et al., 2014). The control variable, *size*, was measured as the number of employees. *Age* was included as a control variable at first but did not have a significant effect on any dependent variable and thus was dropped from the analysis. Table 1 provides an overview of the descriptive statistics for each variable and the item text.

**Table 1:** Overview and descriptive statistics of variables used^a^

	Questions	Mean	Standard deviation	Median
*Dependent variables*				
Resource attraction 1 (RA1)	We are looking specifically for convenient partnerships in order to obtain resources	3.25	1.30	3
Resource attraction 2 (RA2)	We regularly reflect on how we can improve our financing	4.06	1.13	4
Impact focus 1 (IF1)	Our strategy is based on our beliefs about how we can create greater value for our beneficiaries	4.32	0.84	5
Impact focus 2 (IF2)	We define the success of projects and services in terms of beneficiaries’ satisfaction	3.79	0.94	4
Public relations 1 (PR1)	We actively inform public offices about our activities	3.80	1.35	4
Public relations 2 (PR2)	We use public relations to spread information about our organization, projects, or services	3.84	1.36	4
*Independent variables*				
Donations^b^	We seek to acquire money donations from individuals, foundations and/or companies	3.73	1.50	4
Public funding	We seek to acquire public funding	3.31	1.60	4
Earned income	We seek to acquire funding through pricing of our projects and services	3.55	1.49	4
*Control variables*				
Size	Measured as number of employees	62.12	133.69	15
Log(1 + size)		2.81	1.76	2.77

^a^All variables, except *size*, were measured on a five point Likert-scale, ranging from 1 = [I agree] to no extent, to 5 = [I agree] to a large extent

^b^The question on donations was asked in three separate questions asking about each type of donations (from individuals, foundations and companies). Since we are interested in the composed effect we compiled the data using the maximum value from the three responses

**Fig. 3:**
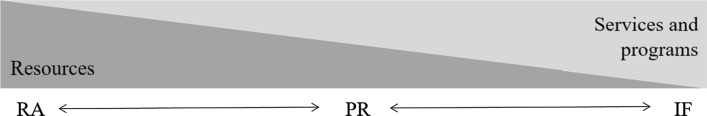
Spectrum of capacity building with specific tasks

As dependent variables, we use selected capacity-building tasks, i.e., tasks directed toward investment in capacities. We focus our analysis on three capacity-building tasks applied across the spectrum described in “The Choice of Investment in Capacities: Utilizing Multitasking Theory” section and depict again in Fig. 3. Capacity-building tasks examined are (1) attracting and securing resources (RA), (2) maintaining public relations (PR), and (3) focusing on the organization’s impact (IF). Although these three tasks by no means cover all possible nonprofit capacities, they cover a broad range of the nonprofit capacity spectrum as introduced by the theoretical framework (chapter 2.2).

Resource attraction is measured from RA1 [adapted from Duque-Zuluaga and Schneider (2008)], which covers partnerships forged to attract resources, both financial and nonfinancial. The second item, RA2, measures the degree to which NPOs are concerned with and want to enhance their financial resources. Public relations items PR1 and PR2 cover two aspects of public relations. PR1 asks about the provision of information to government offices, while PR2 captures the more traditional public relations work of organizations [adapted from Wymer et al. (2015)]. The two impact focus variables, IF1 and IF2, capture the focus of NPOs on their beneficiaries, their most important stakeholder group (Bruce, 1995). These variables are adapted versions of a customer orientation item from Narver and Slater (1990) and the beneficiary orientation item from Modi and Mishra (2010).

The independent variables concern the organizations’ funding intentions and were developed based on statements reflecting support dimensions proposed by Wymer et al. (2015). The categories used (donations, public funding, and earned income) reflect broad funding sources often used to categorize nonprofit finances (von Schnurbein & Fritz, 2017). Financial resources in the form of private donations are a traditional cornerstone of NPO support. However, the share of these sources of financing as a share of total revenue has been declining for some time (Froelich, 1999; Guo, 2006). Support from the public sector, another important resource for NPOs, is also declining in many fields of activity (Boris, 1998). The decline or stagnation of these financing flows is sometimes considered one of the reasons why NPOs are increasingly turning to commercial activities, which enables them to generate their own revenues with which to finance the fulfillment of their core mission (Maier et al., 2016).

The independent variable items measure the intention of an organization to seek funding from this source and not the organization’s actual financing model. The reason for this specification is twofold. First, in terms of data availability, the section of the questionnaire in which NPOs were asked to report their actual financial numbers was left unanswered by more than half of the organizations. Second, the intention to seek financing from a specific funding source allows us to draw conclusions about strategic decisions made in an organization.

### Model

To answer our research question(s), we conducted ordinary least squares (OLS) regressions. The following model was calculated with the six dependent variables (RA1, RA2, PR1, PR2, IF1, and IF2) displayed in Table 1 replacing the dependent variable (DV):

We examined the partial regression plots as recommended by Hair et al. (2014) to assess the linearity of the relationship between the dependent and independent variables of all six regression analyses. Furthermore, partial regression plots help reveal outliers (Hair et al., 2014). Based on the analyses of the partial regression plots, we excluded one observation from the sample due to its high value for variable *size* and applied a logarithmic transformation to this variable. The Breusch–Pagan test is significant for five of the six regressions, indicating the presence of heteroscedasticity. To address this issue, we used a heteroscedasticity-consistent covariance matrix estimation to calculate the standard errors and *p* values of the coefficients (Kleiber & Zeileis, 2008). For the heteroscedasticity-consistent covariance matrix estimation, we followed the approach presented in Fox and Weisberg (2019). The correlation matrix can be found in the appendix (Table 3).

All calculations were performed using R software version 3.6.1 (R Core Team, 2019) using the packages car (Fox & Weisberg, 2019), lmtest (Zeileis & Hothorn, 2002), and sandwich (Zeileis, 2004).

## Results

The results of the six OLS regressions are presented in Table 2. All regressions are significant at the 0.1% significance level. Seeking financial revenue from donations and/or public funding has positive significant effects on investments in capacities related to resource attraction. Additionally, seeking earned income has a positive significant effect on RA2. For RA1, the two significant effects have almost the same effect size (0.191 and 0.194), whereas for RA2, the effect size for donations is higher than that for the other two (0.216 compared to 0.112 and 0.136). The adjusted *R*^2^ values of the two capacity-building tasks referring to resource attraction are 0.27 and 0.21, respectively. Donations and public funding are the two independent variables with significant effects on public relations. Seeking public funding positively affects these capacity-building tasks. Donations, however, have different effects depending on the type of public relations activities engaged therein. Public relations aimed at the general public are positively related to donations (0.293). Meanwhile, NPOs that strongly pursue donations invest significantly less effort (− 0.170) in PR1, which focuses on the provision of information to public offices. Both regressions on public relations capacities resulted in an adjusted *R*^2^ value of higher than 0.25. The two regressions examining the effects of funding goals on impact focus show divergent results. For IF1, the significant variable is donations, while for IF2, earned income is significant. Both regressions have an adjusted *R*^2^ value of below 0.10, which indicates that the independent variables only explain a small proportion of the impact focus capacities.

**Table 2:** Results of OLS regression analyses

*N* = *544*	Resource attraction 1 (RA1)	Resource attraction 2 (RA2)	Impact focus 1 (IF1)	Impact focus 2 (IF2)	Public relations 1 (PR1)	Public relations 2 (PR2)
Donations	0.191*** (0.039)	0.216*** (0.037)	0.107*** (0.028)	0.032 (0.032)	− 0.170*** (0.038)	0.293*** (0.042)
Public funding	0.194*** (0.039)	0.112** (0.035)	− 0.013 (0.025)	0.003 (0.031)	0.374*** (0.036)	0.121** (0.042)
Earned income	0.057 (0.036)	0.136*** (0.034)	0.026 (0.026)	0.120*** (0.029)	0.011 (0.039)	− 0.001 (0.039)
Size (log(1 + *x*))	0.190*** (0.029)	0.037 (0.026)	0.093*** (0.023)	0.000 (0.023)	0.205*** (0.029)	0.220*** (0.032)
Intercept	1.162*** (0.167)	2.292*** (0.188)	3.611*** (0.165)	3.238*** (0.175)	2.582*** (0.216)	1.732*** (0.200)
Multiple *R*^2^	0.27	0.22	0.08	0.04	0.29	0.26
Adjusted *R*^2^	0.27	0.21	0.07	0.03	0.28	0.26
F-statistic	56.50***	30.35***	7.18***	4.90 ***	53.49***	43.29***

^***^0.001, **0.01, *0.05, robust standard errors in parentheses

Control variable *size* has a positive effect on all six variables with four being significant (IF1, RA1, PR1, and PR2). We also calculated the model without control variable *size*. The results are similar, although the adjusted *R*^2^ value is higher for IF1, RA1, PR1 and PR2 when the control variable is added. Only for IF2 does the adjusted *R*^2^ value decrease by 0.01 if the control variable is included. Accordingly, we decided to keep the control variable in the model.

Based on our results, we can confirm the hypothesis formulated as follows: The funding intentions of an NPO influence the effort put into capacity-building tasks, as some tasks are rewarded more than others.

## Discussion

In the present work, we propose a theoretical framework linking the mission and capacities of an organization. Research on the benefits theory has shown that organizations intuitively find funding sources that work best for them and act in accordance with the theory’s predictions (Young, 2017). Additionally, multitasking theory suggests that organizations focus their efforts on tasks that offer the most rewards (Holmstrom & Milgrom, 1991). We therefore assumed the same to be true of the link between funding intentions and investment in capacities. The findings presented in this paper indicate that this second part of the framework, the relationship between funding sources and capacity-building tasks, holds as well. An organization’s purpose therefore implies a certain degree of predetermination that affects not only available funding sources but also the scope for pursuing capacity-building activities.

However, the results show that not all investments in capacities are related to the funding source to the same extent. According to the results in light of the current literature, some aspects seem especially noteworthy. We find support for multitasking theory, as some of the analyzed tasks are influenced more by measurable rewards (funding) than others. The extent of investment in resource attraction and public relations tasks can be explained to a larger degree by the type of funding an organization seeks. It might seem obvious that the two tasks that are closer to the resource side of the capacity-building spectrum react more to the funding sought. For instance, organizations seeking public funding and donations tend to be more concerned with resource-attracting activities. The effect is strongest when seeking donations, as fundraising for private donations requires a great deal of effort and is highly competitive (Ashley & Faulk, 2010; Dolnicar & Lazarevski, 2009).

The lack of explanatory power of funding sources with regard to impact focus, however, is surprising since although impact-related tasks are seemingly positioned further away from funding, they play an increasingly crucial role in attracting funding (Ebrahim, 2003). This result is in line with the recent push for impact measurement in the nonprofit sector with grant-making foundations and watchdog organizations calling for an increased focus on impact (Polonsky & Grau, 2011; von Schnurbein, 2016) and making it a precondition for funding. Not only do private donors want to know what is being done with their money, but public authorities are also under pressure to distribute their funds to effective and legitimate organizations (Suárez, 2011). In the short term, it might seem reasonable to invest in tasks that offer immediate and measurable rewards, such as financial resources. In the long term, however, measuring and reporting on impact is often necessary to secure funding (Arvidson & Lyon, 2014). The results displayed in Table 2 show that the only variable that significantly affects *IF2*, which relates to defining success in terms of beneficiaries’ satisfaction, is seeking earned income. Organizations that aim to finance themselves through earned income are more dependent on satisfied ‘customers’ (Padanyi & Gainer, 2004), and for these organizations, investing in impact-related capacity-building tasks offers a high reward in the form of increased revenue from sales or fees. In the context of multitasking theory, it makes sense for these organizations to invest considerable effort in this task. The results also show that these earned income-reliant organizations seem less concerned with establishing and maintaining partnerships, implying that—compared to other types of NPOs that primarily seek funding through donations or public funds—the demands of their stakeholders may be easier to prioritize.

An explanation for why the regressions on impact focus show low explanatory power might be found in other types of rewards. As mentioned above, the financial rewards of impact measurement are likely to be realized only in the long run. Better reasons for investing in impact focus could include the high intrinsic motivation of nonprofit staff. In this case, stewardship theory (Davis et al., 1997), which takes a positive view of [nonprofit] managers, might be more appropriate for modeling capacity building in this area. Multitasking theory, after all, is a principal-agent theory that considers agents (managers) to be driven by extrinsic motivation only (Holmstrom & Milgrom, 1991).

The results also show that organizations invest significantly less in their relationships with public offices when they heavily seek private donations. This trend could be attributable to a fear of losing independence upon collaborating too closely with government and state institutions, which could lead to a loss of donations (Gazley & Brudney, 2007). The mechanism predicted by multitasking theory is especially evident here, as organizations prioritize tasks (informing the general public) with greater rewards (private donations) at the expense of other tasks (informing public offices) with lesser or even negative rewards. Organizations that seek both donations and public funding may find themselves in a dilemma. Their pursuit of public funding could be improved by providing information to public offices, but this could involve a trade-off resulting in fewer private donations if funders disapprove of close ties with the government. However, this depends on an organization’s type and field of activity; donors might have different expectations of an advocacy organization than of a food bank.

The results emphasize that the mission of an NPO has far-reaching consequences and can result in path dependence, a theoretical construct of organizational research that describes, broadly speaking, “increasingly constrained processes that cannot easily be escaped” (Vergne & Durand, 2010, p. 736). The connection between mission and financing established by the benefits theory can be extended by investment in nonprofit capacities, which are affected by the type of funding an organization seeks. This extension of the benefits theory emphasizes the strong influence of the predetermined organizational purpose. NPOs are therefore prone to finding themselves in a lock-in position (von Schnurbein, 2017). Organizations with a narrow mission are naturally more inclined toward certain capacity-building activities. Although it may benefit them to review their strategies from time to time, they are most likely already tapping into their optimal funding sources, as stated by the benefits theory, as well as putting optimal effort into their capacity building. Organizations with a broader mission theoretically have more options regarding their funding sources and the amount of effort they invest in capacity-building areas and activities. If such organizations want to change their strategic focus, they can benefit from planning ahead and anticipating the amount of effort to dedicate to such activities. Nonprofit managers should be aware of the strong impact of their organizational mission, which can lead to a lock-in status. Strategic considerations should take into account predetermined options with regard to capacity building, especially for organizations with a narrow mission. Larger organizations have a broader scope in terms of capacity-building potential, but strategic thoughts should always start ‘at the top’ of the framework proposed in Fig. 1, i.e., the organization’s mission and how it should be implemented.

## Conclusion

This study analyzed the relationship between funding sources and the amount of effort invested in nonprofit capacities. NPOs often find themselves in a starvation cycle to keep overhead low by focusing more on delivering their services than on having the necessary organizational or financial slack to develop further. It is therefore essential to better understand which factors contribute to NPOs investing in nonprofit capacities in light of the pressures they are subject to and the limited resources available to them. We built our framework on the benefits theory of nonprofit finance, which states that the kind of goods an NPO provides determines which funding sources are available to it. By taking funding sources as predetermined, we extended the influence of this lock-in state to the effort put into capacity-building tasks. We hypothesized that funding is a measurable reward for NPOs and that, in line with multitasking theory, organizations prioritize tasks that offer greater rewards over others.

We tested this framework by analyzing survey data from NPOs in Switzerland and running linear regressions with questions on the effort invested in certain capacities used as dependent variables and those focused on intended funding sources used as independent variables. The results show that seeking a specific funding source affects the amount of effort invested in the analyzed capacity-building tasks. As multitasking theory predicts, tasks that have a measurable reward (funding) are indeed prioritized by organizations, even if not always to the same extent.

The present study is not without limitations. We are aware that the empirical analysis presented in this paper reflects only a first testing of the proposed theoretical framework and that some of the concepts used are simplified and do not do the complexity of reality justice. First, our analysis is limited to a selection of capacity-building tasks. Capacity building can take place through a number of activities, as researchers such as Shumate et al. (2017) have shown, that we did not include in our study. Second, capacity building might also be influenced by factors other than funding and size. External pressures such as watchdog organizations, policies, funders, and competing organizations might also affect investments in certain capacities as well as measures of well-being or the success of an organization such as performance, effectiveness, and organizational slack. Third, since the population of Swiss nonprofits is not known, our study does not claim to be representative of the entire sector. Our results are therefore to be generalized with caution. Fourth, we only consider funding sought (funding intentions) rather than actual funding received. The variables used therefore tell us more about strategic decision-making in organizations than about the actual outcomes of their fundraising efforts. Last, due to issues of data availability, we could not test more elaborate models, for example, with time-lagged variables. We tried to approach the problem of endogeneity with a strong theoretical framework.

Despite these limitations, we identify several avenues for further research resulting from this paper. As a start, researchers could extend the proposed framework by including a performance or effectiveness variable and testing a more complex model through the use of a structural equation model, for instance. From a theoretical perspective, the majority of organizations do not invest effort in capacity-building tasks that are inefficient for them. However, the results presented in this paper do not allow us to draw conclusions on the effectiveness of these tasks. Funding strategy X leading to capacity building Y might not necessarily lead to more successful organizations. Another research opportunity would be to utilize panel data to examine the increasing importance of impact measurement. For now, the ‘reward’ for investing in impact-related capacity building tasks does seem to be of a financial nature, as we do not observe an effect of funding intentions on the extent of investment in these tasks. However, this pattern might change over time, as funders increasingly require organizations to measure and report on impact. It would also be worth more closely examining some of the relationships observed, e.g., the negative effect of seeking donations on providing information to public offices. Some established relationships are not necessarily the result of efficiency but could be due to institutional forces, such as uncertainty, regulation, or professionalization (DiMaggio & Powell, 1983). Analyzing exceptions to our findings (for example, an organization that seeks public funding but does not invest in public relations) could provide deeper insights in this regard. Last, it would be interesting to compare our results, which are based on data from 2018, to results from similar analyses conducted during or after the COVID-19 pandemic. The pandemic is drastically affecting most nonprofits with some not being able to deliver their services and others struggling to find the resources to do so. Future research should analyze how specific funding sources affect efforts invested in capacities differently in light of increased uncertainty and even scarcer resources due to the pandemic. Panel data would offer insights into the effects of COVID-19 on both the funding sources sought and the effort invested in capacities on an organizational level.

This paper lays the groundwork for future research by contributing to the development of various theoretical frameworks. First, this work contributes to the benefits theory of nonprofit finance by extending the well-researched link between mission and funding sources with nonprofit capacities. Second, regarding the theory of path dependency in nonprofits, the results shed light on the strong influence of an organization’s mission on the extent of capacity building through resource attraction, impact focus, and public relations. Third, this work contributes by applying the economic theory of multitasking to the nonprofit context and thereby showing that, in addition to intrinsic motives, measurable rewards in the form of funding play a role in the strategic development of NPOs through capacity building.

## Appendix

See Table 3.

**Table 3:** Correlations between variables used

		1	2	3	4	5	6	7	8	9	10
1	IF1	1.000									
2	IF2	0.199	1.000								
3	RA1	0.178	0.082	1.000							
4	RA2	0.273	0.120	0.346	1.000						
5	PR1	0.123	0.140	0.220	0.154	1.000					
6	PR2	0.278	0.063	0.413	0.281	0.264	1.000				
7	Donations	**0.186**	**0.069**	**0.332**	**0.370**	**0.005**	**0.387**	*1.000*			
8	Public funding	**0.112**	**0.079**	**0.410**	**0.345**	**0.425**	**0.343**	*0.429*	*1.000*		
9	Earned income	**0.106**	**0.195**	**0.218**	**0.262**	**0.190**	**0.140**	*0.082*	*0.277*	*1.000*	
10	Log(1 + size)	**0.202**	**0.052**	**0.330**	**0.143**	**0.366**	**0.318**	*0.009*	*0.218*	*0.265*	*1.000*

Italic numbers show the correlation between the independent variables, the bold numbers show the correlations between the independent and dependent variables, the gray numbers show the correlations between the dependent variables
